# MicroRNAs and Osteolytic Bone Metastasis: The Roles of MicroRNAs in Tumor-Induced Osteoclast Differentiation

**DOI:** 10.3390/jcm4091741

**Published:** 2015-08-28

**Authors:** Tadayoshi Kagiya

**Affiliations:** Division of Functional Morphology, Department of Anatomy, Iwate Medical University, 2-1-1 Nishitokuta, Yahaba-cho, Iwate, 028-3694, Japan; E-Mail: tkagiya@iwate-med.ac.jp; Tel.: +81-19-651-5111; Fax: +81-19-908-8010.

**Keywords:** Bone Metastasis, Osteoclasts, MicroRNAs, Exosomes, Extracellular Vesicles

## Abstract

Osteolytic bone metastasis frequently occurs in the later stages of breast, lung, and several other cancers. Osteoclasts, the only cells that resorb bone, are hijacked by tumor cells, which break down bone remodeling systems. As a result, osteolysis occurs and may cause patients to suffer bone fractures, pain, and hypercalcemia. It is important to understand the mechanism of bone metastasis to establish new cancer therapies. MicroRNAs are small, noncoding RNAs that are involved in various biological processes, including cellular differentiation, proliferation, apoptosis, and tumorigenesis. MicroRNAs have significant clinical potential, including their use as new therapeutic targets and disease-specific biomarkers. Recent studies have revealed that microRNAs are involved in osteoclast differentiation and osteolytic bone metastasis. In this review focusing on microRNAs, the author discusses the roles of microRNAs in osteoclastogenesis and osteolytic bone metastasis.

## 1. Introduction 

Cancer is one of the most common causes of death, and bone is the third most common cancer metastatic site following the lung and liver [1]. Patients with metastasis to bone often present with lesions that can be osteoblastic, osteolytic, or a mixture of the two [2]. These lesions result from an imbalance between osteoblastic bone formation and osteoclastic bone resorption. Osteoblastic bone metastasis is caused by excessive osteoblast activity relative to osteoclast activity, a characteristic of prostate cancer [2]. In contrast, osteolytic bone metastasis is caused by excessive osteoclast activity relative to osteoblast activity [2]. Osteolytic bone metastasis frequently occurs in the later stages of breast, lung, and several other cancers [2,3]. Osteoclasts are hijacked by tumor cells, which break down bone remodeling systems [3,4]. As a result, osteolysis occurs and may cause patients to suffer bone fractures, pain, and hypercalcemia [3,4]. Thus, the quality of life of patients is negatively affected.

**Figure 1:**
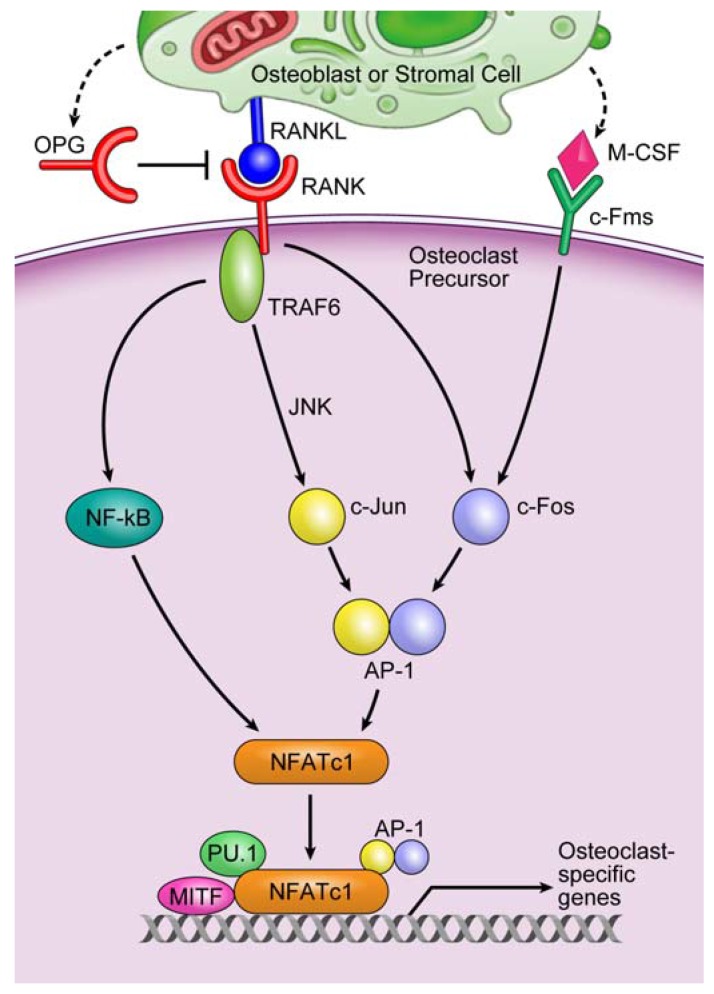
An important signaling cascade of osteoclastogenesis. The binding of M-CSF to its receptor, c-Fms, induces the transcription factor c-Fos, whereas the binding of RANKL to its receptor, RANK, leads to the recruitment of TRAF6, the main adapter molecule of RANK. TRAF6 activates NF-κB and mitogen-activated kinases including JNK. JNK in turn activates the transcription factor c-Jun. RANKL/RANK also induces c-Fos to form AP-1, a heterodimeric transcription factor, with c-Jun. AP-1 and NF-κB then induce NFATc1, a master transcription factor that regulates osteoclast differentiation. NFATc1 works together with other transcription factors such as AP-1, PU.1, and MITF to induce various osteoclast-specific genes. Thus, M-CSF and RANKL signaling pathways are crucial for osteoclastogenesis. On the other hand, the RANK–RANKL interaction is inhibited by the decoy receptor OPG expressed by stromal cells and osteoblasts.

Osteoclasts are the only cells that resorb bone [5]. Osteoclasts are tartrate-resistant acid phosphatase (TRAP)-positive multinucleated giant cells [5,6,7], and are formed by the fusion of hematopoietic cells of the monocyte/macrophage lineage. Although osteoclastogenesis is regulated by a variety of hormones, growth factors, and cytokines, macrophage colony-stimulating factor (M-CSF) and receptor activator of nuclear factor κB ligand (RANKL), which are expressed in stromal cells and osteoblasts, are essential for osteoclast differentiation [5,6]. The binding of M-CSF to its receptor, c-Fms, induces the transcription factor c-Fos, whereas the binding of RANKL to its receptor, receptor activator of nuclear factor κB (RANK), leads to the recruitment of TNF-receptor-associated factor 6 (TRAF6), the main adapter molecule of RANK. TRAF6 activates nuclear factor κB (NF-κB) and mitogen-activated kinases, including c-Jun N-terminal kinase (JNK). JNK in turn activates the transcription factor c-Jun [8]. RANKL/RANK also induces c-Fos to form activator protein-1 (AP-1), a heterodimeric transcription factor, with c-Jun. AP-1 and NF-κB then induce nuclear factor of activated T cell cytoplasmic 1 (NFATc1), a master transcription factor that regulates osteoclast differentiation. NFATc1 works together with other transcription factors such as AP-1, PU.1, and microphthalmia-associated transcription factor (MITF) to induce various osteoclast-specific genes [8]. Thus, M-CSF and RANKL signaling pathways are crucial for osteoclastogenesis. In contrast, the RANK–RANKL interaction is inhibited by the decoy receptor osteoprotegerin (OPG), a soluble member of the TNF receptor superfamily expressed by stromal cells and osteoblasts [9,10] (Figure 1). Thus, osteoclastogenesis is appropriately regulated in normal physiological conditions.

## 2. Bone Metastasis

Metastasis to bone is mainly blood-borne [1,9,11,12]. Tumor cells first detach from the primary lesion and invade the blood vessels. Once in the bloodstream, tumor cells are attracted to preferred sites of metastasis through site-specific interactions between tumor cells and cells in the target organ [1,11]. To metastasize, a tumor cell must gain access to the vasculature from the primary tumor, survive the circulation, escape immune surveillance, and localize in the vasculature of the target organ [1,13]. Most single or clustered tumor cells are thought to expire in the circulation and fail to metastasize because of either anoikis, mechanical trauma, or attack and clearance by the host defense system [13]. Although circulating tumor cells have been hypothesized to persist as single cells or small cell clusters, there is another pathway of blood-borne metastasis [13,14,15,16]. Tumor nets are enveloped by vascular endothelial cells and enter the circulation, and tumor emboli may form [13,14,15]. Tumor emboli are composed of multicellular tumor nets that are sufficiently large enough to arrest in the target organ, where they thrive and create expansive secondary tumors [13,14,15]. In patients with hepatocellular carcinoma, the tumor emboli conserve elements of their primary tumor tissue organization, and are associated with the basement membrane and vascular endothelial cells on the surface [13]. This architecture can provide an integrated ecosystem that protects the tumor cells from anoikis, mechanical trauma, and immunological engagement during dissemination [13]. Whether this “invasion-independent metastasis” is involved in bone metastasis is unknown and, considering the abundance of blood in bone tissue, there is a possibility of invasion-independent bone metastasis.

Once tumor cells that metastasize to the skeleton reach the bone marrow, they interact with anatomical entities in contact with the bone called niches. Two different niches exist: the endosteal niche, where stem cells are closely associated with stromal cells and osteoblasts, and the vascular niche, where hematopoietic cells are located [12]. 

## 3. Microenvironment of Osteolytic Lesions

### 3.1. Growth Factors in the Microenvironment of Osteolytic Lesions

**Figure 2:**
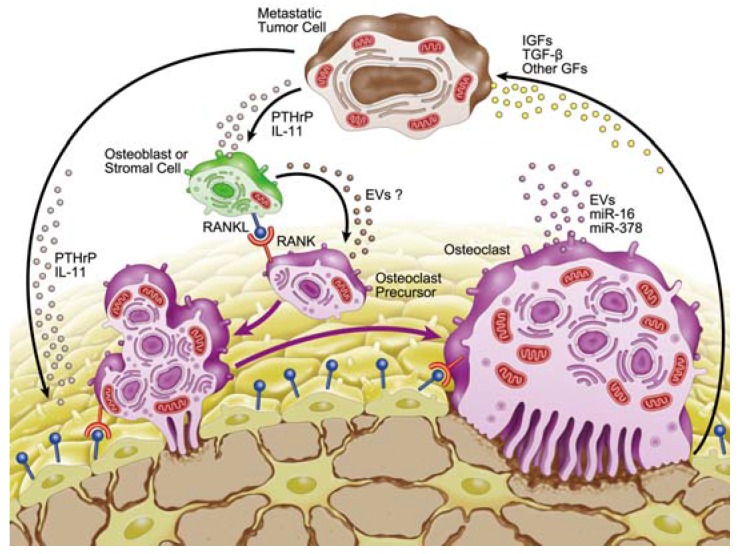
Schematic view of tumor-induced osteoclast formation. Bone-derived growth factors (IGFs, TGF-β and other growth factors) are released by osteoclastic bone resorption. These factors promote tumor cell proliferation and survival. TGF-β acts on tumor cells to produce growth factors, such as PTHrP and IL-11. PTHrP acts on osteoblasts and stromal cells and promotes the expression of RANKL, resulting in the enhancement of osteoclastogenesis and destruction of bone. Osteoclasts secrete extracellular vesicles (EVs) containing specific microRNAs, such as miR-21, miR-210, and miR-378. miR-16 and miR-378 are secreted biomarkers for osteolytic bone metastasis.

The bone microenvironment comprises osteoblasts, stromal cells, osteoclasts, mineralized bone matrix, hematopoietic cells, and many other cell types [11]. Bone matrix contains a variety of growth factors, such as insulin-like growth factors (IGFs), transforming growth factor β (TGF-β), fibroblast growth factors, platelet-derived growth factors, and bone morphogenetic proteins [1,2,9,11,12,17,18]. These bone-derived growth factors are released by osteoclastic bone resorption, and colonization of tumor cells in bone is under the influence of these growth factors. For example, TGF-β is one of the most abundant growth factors in bone matrix [11]. TGF-β released from bone matrix inhibits T-cell proliferation and activity and the function of natural killer cells, thereby suppressing the immune system [17]. In addition, TGF-β promotes tumor cell proliferation and survival [1]. In breast cancer, TGF-β released from the matrix as a result of increased bone resorption can cause tumor cells to produce growth factors such as parathyroid hormone-related protein (PTHrP) and interleukin 11 (IL-11) that can perturb the RANKL/OPG balance, resulting in further osteoclastogenesis and perpetuation of osteolytic disease [2] (Figure 2).

PTHrP is one of the most important mediators of osteoclast activation [1,2,9,11,12,17,18,19]. More than 90% of patients with breast cancer that has metastasized to bone overexpress PTHrP [17,19]. In addition, PTHrP expression has been determined to be a risk factor for predicting bone metastasis in patients with breast cancer [2]. In the bone microenvironment, PTHrP is produced by osteoblasts, stromal cells, and cancer cells [1,2,9,11,12,17,18,19]. PTHrP acts on osteoblasts and stromal cells and promotes cellular expression of RANKL, resulting in enhancement of osteoclastogenesis and destruction of bone [2,9,11,17,18,19]. Consequently, growth factors are further released from resorbing bone and promote colonization of metastatic tumor cells in bone [1,2,9,11,17,18,19]. This represents a “vicious circle” between metastatic tumor cells and bone cells (Figure 2). 

### 3.2. Involvement of microRNAs in Tumor-Induced Osteoclast Differentiation

It was recently revealed that microRNAs (miRNAs) play important roles in tumorigenesis and tumor progression [20]. miRNAs are small, endogenous, noncoding RNAs of approximately 20 to 22 nucleotides in length [3,5,6,20]. Although the biological functions of most miRNAs are not yet fully understood, they participate in the regulation of cellular differentiation, proliferation, apoptosis, and cancer development [5,6,20]. Transcription of miRNA genes yields noncoding transcripts that are subsequently processed through sequential digestion by the RNase III enzymes Drosha and Dicer [5,6,20]. The resulting single-stranded mature miRNAs are finally incorporated into an RNA-induced silencing complex (RISC) that contains argonaute (Ago) family proteins [5,6,20]. The Ago proteins recruit miRNAs specific to the target mRNAs, and the RISC inhibits the translation of target mRNAs and/or degrades target mRNAs. Thus, miRNAs are involved in post-transcriptional regulation of mRNA function [5,6,20].

Recent studies have revealed that miRNAs play critical roles in osteoclastogenesis. We reported that the expression of 52 mature miRNAs differed more than two-fold between untreated cells and cells treated with RANKL during osteoclastogenesis [5]. As a key factor in osteoclast differentiation, miR-223 regulates nuclear factor I-A and M-CSF receptor levels [21,22]. miR-124 regulates osteoclastogenesis by suppressing NFATc1, a master transcription factor of osteoclast differentiation [23]. RANKL-induced c-Fos upregulates miR-21, which downregulates the expression of programmed cell death 4 (PDCD4), a negative regulator of osteoclastogenesis [24]. Overexpression of miR-155 blocks osteoclast differentiation by repressing MITF and PU.1, which are crucial transcription factors for osteoclast differentiation [25]. These reports are based on murine cell experiments; recent work has begun to uncover the roles of miRNAs in human osteoclast differentiation and function. miR-29b negatively regulates human osteoclastic cell differentiation and function by suppressing c-Fos [26]. The expression level of miR-503, which directly targets RANK, is markedly lower in progenitors of osteoclasts from postmenopausal women with osteoporosis than in those from postmenopausal healthy women [27]. The repressive effects on monocyte-specific genes by let-7e/miR-99b/125a/132/212 are crucial for human osteoclast differentiation. These miRNAs are activated directly by NF-κB and exhibit rapid upregulation during osteoclast differentiation. Their inhibition impairs osteoclastogenesis [28].

It was recently revealed that miRNAs are involved in tumor-induced osteoclast differentiation (Table 1). Ell *et al.* [3] reported that five miRNAs (miR-33a, miR-133a, miR-141, miR-190, and miR-219) are significantly downregulated during osteoclastogenesis in both normal physiological conditions and pathophysiological cancer conditions. Ectopic expression of miR-133a, miR-141, and miR-219 strongly inhibited osteoclast differentiation and bone resorption by directly targeting *Mitf/Mmp14*, *Mitf/Calcr*, and *Mitf/Traf6*, respectively. Remarkably, miR-141 and miR-219 administered systemically led to a significant decrease in the number of osteoclasts *in vivo,* and also reduced the metastatic tumor burden in an experimental breast cancer model [3]. Krzezinski *et al.* [29] reported that miR-34a blocks osteoporosis and bone metastasis by inhibiting osteoclastogenesis. The expression level of miR-34a decreases during osteoclastogenesis, and knockdown of miR-34a promotes osteoclast differentiation, while ectopic miR-34a inhibits this differentiation. They also identified transforming growth factor β-induced factor 2 (Tgif2), which is induced by NFATc1 and AP-1 during osteoclast differentiation, as a direct target of miR-34a. miR-34a plays important roles in osteoblast and osteoclast differentiation. Osteoblast differentiation is reduced in miR-34a knockout mice but increased in osteoblastic miR-34a conditional transgenic mice [29].

The involvement of miRNAs in growth factors of osteolytic lesions has also been shown. TGF-β is released from bone matrix during osteoclastic bone resorption and induces cancer cells to produce osteolytic factors such as IL-11 [30]. Three miRNAs (miR-204, miR-211, and miR-379) inhibit TGF-β-induced IL-11 production in bone metastatic breast cancer cells [30]. Kuo *et al*. [31] reported that miR-33a functions as a bone metastasis suppressor in lung cancer by targeting *PTHrP*. miR-33a is downregulated in lung cancer cells, which express high levels of PTHrP. PTHrP enhances osteoclastogenesis by altering the ratio of osteoclastogenesis activator (M-CSF and RANKL)/inhibitor (OPG) produced by osteoblasts. Ectopic miR-33a decreases the induction of lung cancer cells in the production of M-CSF and RANKL in osteoblasts and increases that of OPG in osteoblasts by suppressing PTHrP [31].

Collectively, specific miRNAs play critical roles in osteoclastogenesis under normal physiological conditions and in tumor-induced osteoclast differentiation. 

## 4. Involvement of Extracellular Vesicles in Osteolytic Bone Metastasis

miRNAs were recently reported to be present in exosomes [41], a kind of extracellular vesicle (EV), and to function in other cells [37,42]. EVs are lipid bilayered vesicles that exist outside of cells. There are three main types of EVs: apoptotic bodies, microvesicles, and exosomes. Apoptotic bodies are 800 to 5000 nm in diameter and are released by apoptotic cells. Microvesicles are 50 to 1000 nm in diameter and are formed by budding directly from the plasma membrane. Exosomes are 40 to 100 nm in diameter and are derived from multivesicular bodies [43,44,45]. Two principal methods of collecting EVs are currently used: with and without ultracentrifugation [6,45]. However, the techniques are inadequate for collecting each type of EV [44,45]. Considering this fact and that the nomenclature of exosomes is confusing [46], this paper does not use the term “exosomes,” but rather “EVs”. EVs have an important role in cell-to-cell communication via the transfer of miRNAs, mRNAs, proteins, and bioactive lipids to target cells [6,37,41,42,44,47]. The secretion of EVs containing miRNAs depends on the cell type, biological condition, and types of miRNAs the cells contain [6,47].

**Table 1:** Selected miRNAs important for progression of osteolytic metastasis.

miRNA	Function(s)	Reference(s)
miR-16	Potential circulating biomarker for bone metastasis	[3]
miR-21	Functions as an oncogene	[32]
	Highly expressed during osteoclastogenesis	[24]
	Highly detected in osteoclast EVs	[6]
miR-31	Inhibits breast cancer metastasis	[33]
	Promotes ring-shaped mature osteoclast formation	[34]
miR-33a	Inhibits bone metastasis by targeting *PTHrP*	[31]
	Downregulated during osteoclastogenesis	[3]
miR-34a	Inhibits osteoclast differentiation by targeting *Tgif2*	[29]
	Attenuates bone metastasis	[29]
miR-125a	Tumor suppressor in breast cancer	[32]
	Upregulated during osteoclastogenesis	[28]
	Inhibits osteoclast differentiation by targeting *TRAF6*	[35]
miR-133a	Inhibits osteoclast differentiation by targeting *Mitf* and *Mmp14*	[3]
miR-141	Inhibits osteoclast differentiation by targeting *Mitf* and *Calcr*	[3]
miR-155	Highly expressed in invasive tumors	[32]
	Inhibits osteoclastogenesis by repressing MITF and PU.1	[25]
	Deficiency promotes tumor growth *in vivo*	[36]
miR-190	Inhibits osteoclast differentiation by targeting *Calcr*	[3]
miR-192	Inhibits angiogenesis and decreases bone metastasis	[37]
miR-219	Inhibits osteoclast differentiation by targeting *Mitf* and *Traf6*	[3]
miR-223	Inhibits murine osteoclast differentiation	[21,22]
	Decreases breast cancer cell proliferation	[38]
miR-326	Potential circulating biomarker for bone metastasis	[39]
miR-378	Potential circulating biomarker for bone metastasis	[3]
	Highly detected in osteoclast EVs	[6]
	Promotes cell survival, tumor growth, and angiogenesis	[40]
miR-204/211/379	Inhibits TGF-β-induced IL-11 production	[30]

Although osteoclasts play important roles in osteolytic bone metastasis, whether osteoclasts secrete EVs containing miRNAs was unknown until recently. Therefore, we investigated eight miRNAs in the EVs deemed important for osteoclastogenesis in our previous study: let-7e, miR-21, miR-33, miR-155, miR-210, miR-223, miR-378, and miR-1224 [6]. Of these, the expression levels of miR-378, miR-21, and miR-210 were very high, while no significant expression of miR-33 or miR-1224 was detected [6]. These results suggest that osteoclasts secrete EVs containing specific miRNAs, but that they do not contain the entire set of intracellular miRNAs. miR-16 and miR-378 are reportedly higher in serum from mice with highly metastatic breast cancer cells and in serum from patients with breast cancer metastasis to bone than in healthy female donors [3]. miRNAs in serum and plasma are divided into two populations: a vesicle-associated membrane-bound form and a ribonucleoprotein-associated non-membrane-bound form [48]. Considering that most miR-16 in human serum is present in the ribonucleoprotein-associated non-membrane-bound form [48], increased levels of miR-16 in the serum of patients with bone metastasis may be of the ribonucleoprotein-associated non-membrane-bound form. Valencia *et al*. [39] reported that serum miR-326 could potentially serve as a novel biochemical marker for monitoring bone metastasis from lung cancer. They reported that the level of miR-326 may not only reflect tumor-autonomous release, but also host-derived factors acting on tumor cells, because miR-326 has been implicated in lymphocytic differentiation, chemoresistance, and tumor-suppressive activities [39]. 

While these reports are important, the function of miRNAs and EVs was not mentioned. Several reports suggest the involvement of EVs in osteolytic bone metastasis. One suggests that forced expression of miR-192 in EVs of highly metastatic lung cancer cells decreases osteolytic lesions in a mouse model. miR-192 in the EVs is transferred to endothelial cells and inhibits angiogenesis [37]. A second study showed that EVs from multiple myeloma cells increase CXC-chemokine receptor 4 expression in pre-osteoclasts and modulate cell migration. EVs derived from the serum of patients with multiple myeloma promote osteoclast differentiation [49]. A third report showed that EVs from parathyroid hormone (PTH)-treated UAMS-32P cells from a stromal/osteoblastic cell line promote osteoclast differentiation. The EVs containing RANK, RANKL receptor, and RANKL antibody treatment inhibited osteoclastogenesis [50]. Thus, EVs from PTH-treated osteoblastic cells promote osteoclast differentiation via RANK/RANKL signaling. Given that both PTH and PTHrP bind to the same receptor, the PTH/PTHrP receptor, PTHrP from tumor cells may stimulate stromal/osteoblastic cells to secrete EVs, and thus the EVs may induce osteoclast differentiation (Figure 2).

## 5. Conclusions

Bone metastasis is a highly complicated process, and the bone microenvironment contains numerous physical factors. Although a single miRNA generally represses the production of hundreds of proteins, the repression is typically mild [51]. Considering this mild effect, it may be necessary to combine miRNA-based and traditional routine therapies to successfully treat bone metastasis. For example, combination treatments with miRNAs and currently approved osteoclast-targeting agents, such as bisphosphonates and the anti-RANKL antibody denosumab, might provide enhanced clinical efficiency. Although it may be a long way to the use of miRNAs as therapeutic agents, we anticipate that this new therapeutic target for bone metastasis opens another door to cancer treatment.

## References

[1] Krzeszinski J.Y., Wan Y. (2015). New therapeutic targets for cancer bone metastasis. Trends Pharmacol. Sci..

[2] Browne G., Taipaleenmaki H., Stein G.S., Stein J.L., Lian J.B. (2014). MicroRNAs in the control of metastatic bone disease. Trends Endocrinol. Metab..

[3] Ell B., Mercatali L., Ibrahim T., Campbell N., Schwarzenbach H., Pantel K., Amadori D., Kang Y. (2013). Tumor-induced osteoclast miRNA changes as regulators and biomarkers of osteolytic bone metastasis. Cancer Cell.

[4] Waning D.L., Mohammad K.S., Guise T.A. (2013). Cancer-associated osteoclast differentiation takes a good look in the miR(NA)ror. Cancer Cell.

[5] Kagiya T., Nakamura S. (2013). Expression profiling of microRNAs in RAW264.7 cells treated with a combination of tumor necrosis factor alpha and RANKL during osteoclast differentiation. J. Periodontal Res..

[6] Kagiya T., Taira M. (2013). Expression of MicroRNAs in the Extracellular Microvesicles of Murine Osteoclasts. J. Oral Tissue Engin..

[7] Itzstein C., Coxon F.P., Rogers M.J. (2011). The regulation of osteoclast function and bone resorption by small GTPases. Small GTPases.

[8] Nakashima T., Takayanagi H. (2009). Osteoclasts and the immune system. J. Bone Miner. Metab..

[9] Weilbaecher K.N., Guise T.A., McCauley L.K. (2011). Cancer to bone: a fatal attraction. Nat. Rev. Cancer.

[10] Jones D.H., Nakashima T., Sanchez O.H., Kozieradzki I., Komarova S.V., Sarosi I., Morony S., Rubin E., Sarao R., Hojilla C.V. (2006). Regulation of cancer cell migration and bone metastasis by RANKL. Nature.

[11] Kingsley L.A., Fournier P.G., Chirgwin J.M., Guise T.A. (2007). Molecular biology of bone metastasis. Mol. Cancer Ther..

[12] Croset M., Santini D., Iuliani M., Fioramonti M., Zoccoli A., Vincenzi B., Tonini G., Pantano F. (2014). MicroRNAs and bone metastasis: a new challenge. Molecules.

[13] Sugino T., Yamaguchi T., Hoshi N., Kusakabe T., Ogura G., Goodison S., Suzuki T. (2008). Sinusoidal tumor angiogenesis is a key component in hepatocellular carcinoma metastasis. Clin. Exp. Metastasis.

[14] Sugino T., Kusakabe T., Hoshi N., Yamaguchi T., Kawaguchi T., Goodison S., Sekimata M., Homma Y., Suzuki T. (2002). An invasion-independent pathway of blood-borne metastasis: a new murine mammary tumor model. Am. J. Pathol..

[15] Kats-Ugurlu G., Roodink I., de Weijert M., Tiemessen D., Maass C., Verrijp K., van der Laak J., de Waal R., Mulders P., Oosterwijk E. (2009). Circulating tumour tissue fragments in patients with pulmonary metastasis of clear cell renal cell carcinoma. J. Pathol..

[16] Al-Mehdi A.B., Tozawa K., Fisher A.B., Shientag L., Lee A., Muschel R.J. (2000). Intravascular origin of metastasis from the proliferation of endothelium-attached tumor cells: a new model for metastasis. Nat. Med..

[17] Suva L.J., Washam C., Nicholas R.W., Griffin R.J. (2011). Bone metastasis: mechanisms and therapeutic opportunities. Nat. Rev. Endocrinol..

[18] Hiraga T., Myoui A., Hashimoto N., Sasaki A., Hata K., Morita Y., Yoshikawa H., Rosen C.J., Mundy G.R., Yoneda T. (2012). Bone-derived IGF mediates crosstalk between bone and breast cancer cells in bony metastases. Cancer Res..

[19] Martin T.J. (2002). Manipulating the environment of cancer cells in bone: a novel therapeutic approach. J. Clin. Invest..

[20] Takahashi R.U., Miyazaki H., Ochiya T. (2015). The Roles of MicroRNAs in Breast Cancer. Cancers.

[21] Sugatani T., Hruska K.A. (2007). MicroRNA-223 is a key factor in osteoclast differentiation. J. Cell Biochem..

[22] Sugatani T., Hruska K.A. (2009). Impaired micro-RNA pathways diminish osteoclast differentiation and function. J. Biol. Chem..

[23] Lee Y., Kim H.J., Park C.K., Kim Y.G., Lee H.J., Kim J.Y., Kim H.H. (2013). MicroRNA-124 regulates osteoclast differentiation. Bone.

[24] Sugatani T., Vacher J., Hruska K.A. (2011). A microRNA expression signature of osteoclastogenesis. Blood.

[25] Mann M., Barad O., Agami R., Geiger B., Hornstein E. (2010). miRNA-based mechanism for the commitment of multipotent progenitors to a single cellular fate. Proc. Natl. Acad. Sci. USA.

[26] Rossi M., Pitari M.R., Amodio N., Di Martino M.T., Conforti F., Leone E., Botta C., Paolino F.M., Del Giudice T., Iuliano E. (2013). miR-29b negatively regulates human osteoclastic cell differentiation and function: implications for the treatment of multiple myeloma-related bone disease. J. Cell Physiol..

[27] Chen C., Cheng P., Xie H., Zhou H.D., Wu X.P., Liao E.Y., Luo X.H. (2014). MiR-503 regulates osteoclastogenesis via targeting RANK. J. Bone Miner. Res..

[28] De la Rica L., Garcia-Gomez A., Comet N.R., Rodriguez-Ubreva J., Ciudad L., Vento-Tormo R., Company C., Alvarez-Errico D., Garcia M., Gomez-Vaquero C. (2015). NF-kappaB-direct activation of microRNAs with repressive effects on monocyte-specific genes is critical for osteoclast differentiation. Genome Biol..

[29] Krzeszinski J.Y., Wei W., Huynh H., Jin Z., Wang X., Chang T.C., Xie X.J., He L., Mangala L.S., Lopez-Berestein G. (2014). miR-34a blocks osteoporosis and bone metastasis by inhibiting osteoclastogenesis and Tgif2. Nature.

[30] Pollari S., Leivonen S.K., Perala M., Fey V., Kakonen S.M., Kallioniemi O. (2012). Identification of microRNAs inhibiting TGF-beta-induced IL-11 production in bone metastatic breast cancer cells. PLoS One.

[31] Kuo P.L., Liao S.H., Hung J.Y., Huang M.S., Hsu Y.L. (2013). MicroRNA-33a functions as a bone metastasis suppressor in lung cancer by targeting parathyroid hormone related protein. Biochim. Biophys. Acta..

[32] O’Day E., Lal A. (2010). MicroRNAs and their target gene networks in breast cancer. Breast Cancer Res..

[33] Valastyan S., Weinberg R.A. (2010). miR-31: a crucial overseer of tumor metastasis and other emerging roles. Cell Cycle.

[34] Mizoguchi F., Murakami Y., Saito T., Miyasaka N., Kohsaka H. (2013). miR-31 controls osteoclast formation and bone resorption by targeting RhoA. Arthritis Res. Ther..

[35] Guo L.J., Liao L., Yang L., Li Y., Jiang T.J. (2014). MiR-125a TNF receptor-associated factor 6 to inhibit osteoclastogenesis. Exp. Cell Res..

[36] Wang J., Yu F., Jia X., Iwanowycz S., Wang Y., Huang S., Ai W., Fan D. (2015). MicroRNA-155 deficiency enhances the recruitment and functions of myeloid-derived suppressor cells in tumor microenvironment and promotes solid tumor growth. Int. J. Cancer.

[37] Valencia K., Luis-Ravelo D., Bovy N., Anton I., Martinez-Canarias S., Zandueta C., Ormazabal C., Struman I., Tabruyn S., Rebmann V. (2014). miRNA cargo within exosome-like vesicle transfer influences metastatic bone colonization. Mol. Oncol..

[38] Lim P.K., Bliss S.A., Patel S.A., Taborga M., Dave M.A., Gregory L.A., Greco S.J., Bryan M., Patel P.S., Rameshwar P. (2011). Gap junction-mediated import of microRNA from bone marrow stromal cells can elicit cell cycle quiescence in breast cancer cells. Cancer Res..

[39] Valencia K., Martin-Fernandez M., Zandueta C., Ormazabal C., Martinez-Canarias S., Bandres E., de la Piedra C., Lecanda F. (2013). miR-326 associates with biochemical markers of bone turnover in lung cancer bone metastasis. Bone.

[40] Lee D.Y., Deng Z., Wang C.H., Yang B.B. (2007). MicroRNA-378 promotes cell survival, tumor growth, and angiogenesis by targeting SuFu and Fus-1 expression. Proc. Natl Acad Sci USA.

[41] Valadi H., Ekstrom K., Bossios A., Sjostrand M., Lee J.J., Lotvall J.O. (2007). Exosome-mediated transfer of mRNAs and microRNAs is a novel mechanism of genetic exchange between cells. Nat. Cell. Biol..

[42] Kosaka N., Iguchi H., Yoshioka Y., Takeshita F., Matsuki Y., Ochiya T. (2010). Secretory mechanisms and intercellular transfer of microRNAs in living cells. J. Biol. Chem..

[43] Carandini T., Colombo F., Finardi A., Casella G., Garzetti L., Verderio C., Furlan R. (2015). Microvesicles: What is the Role in Multiple Sclerosis?. Front Neurol..

[44] Nishida-Aoki N., Ochiya T. (2015). Interactions between cancer cells and normal cells via miRNAs in extracellular vesicles. Cell. Mol. Life Sci..

[45] Crescitelli R., Lasser C., Szabo T.G., Kittel A., Eldh M., Dianzani I., Buzas E.I., Lotvall J. (2013). Distinct RNA profiles in subpopulations of extracellular vesicles: apoptotic bodies, microvesicles and exosomes. J. Extracell Vesicles.

[46] Gould S.J., Raposo G. (2013). As we wait: coping with an imperfect nomenclature for extracellular vesicles. J. Extracell Vesicles.

[47] Kagiya T., Taira M., Rogers J.V. (2014). A New Application for Microarrays: Analysis of Global MicroRNA Expression Profiles in the Extracellular Microvesicles of Human Macrophage-like Cells. Microarrays: Principles, Applications and Technologies.

[48] Arroyo J.D., Chevillet J.R., Kroh E.M., Ruf I.K., Pritchard C.C., Gibson D.F., Mitchell P.S., Bennett C.F., Pogosova-Agadjanyan E.L., Stirewalt D.L. (2011). Argonaute2 complexes carry a population of circulating microRNAs independent of vesicles in human plasma. Proc. Natl. Acad. Sci. USA.

[49] Raimondi L., De Luca A., Amodio N., Manno M., Raccosta S., Taverna S., Bellavia D., Naselli F., Fontana S., Schillaci O. (2015). Involvement of multiple myeloma cell-derived exosomes in osteoclast differentiation. Oncotarget.

[50] Deng L., Wang Y., Peng Y., Wu Y., Ding Y., Jiang Y., Shen Z., Fu Q. (2015). Osteoblast-derived microvesicles: A novel mechanism for communication between osteoblasts and osteoclasts. Bone.

[51] Selbach M., Schwanhausser B., Thierfelder N., Fang Z., Khanin R., Rajewsky N. (2008). Widespread changes in protein synthesis induced by microRNAs. Nature.

